# Preoperative intra-aortic balloon pump to reduce mortality in coronary artery bypass graft: a meta-analysis of randomized controlled trials

**DOI:** 10.1186/s13054-014-0728-1

**Published:** 2015-01-14

**Authors:** Alberto Zangrillo, Federico Pappalardo, Roberto Dossi, Ambra Licia Di Prima, Marta Eugenia Sassone, Teresa Greco, Fabrizio Monaco, Mario Musu, Gabriele Finco, Giovanni Landoni

**Affiliations:** Department of Anaesthesia and Intensive Care, IRCCS San Raffaele Scientific Institute, Via Olgettina 60, Milan, 20132 Italy; Vita-Salute University San Raffaele of Milan, Via Olgettina, 58, 20132 Milan, Italy; Department of Medical Sciences “M. Aresu”, Cagliari University, Bivio di Sestu 554, 09042 Monserrato, Cagliari Italy

## Abstract

**Introduction:**

The intra-aortic balloon pump is routinely used in cardiac surgery; however, its impact on outcome is still a matter of debate and several randomized trials have been published recently. We perform an updated meta-analysis of randomized controlled trials that investigated the use of preoperative intra-aortic balloon pump in adult patients undergoing coronary artery bypass grafting.

**Methods:**

Potentially eligible trials were identified by searching the Medline, Embase, Scopus, ISI Web of Knowledge and The Cochrane Library. Searches were not restricted by language or publication status and were updated in August 2014. Randomized controlled trials on preoperative intra-aortic balloon pump in patients undergoing coronary artery bypass grafting either with or without cardiopulmonary bypass were identified. The primary end point was mortality at the longest follow-up available and the secondary end point was 30-day mortality.

**Results:**

The eight included randomized clinical trials enrolled 625 patients (312 to the intra-aortic balloon pump group and 313 to control). The use of intra-aortic balloon pump was associated with a significant reduction in the risk of mortality (11 of 312 (3.5%) versus 33 of 313 (11%), risk ratio = 0.38 (0.20 to 0.73), *P* for effect = 0.004, *P* for heterogeneity = 0.7, I-square = 0%, with eight studies included). The benefit on mortality reduction was confirmed restricting the analysis to trials with low risk of bias, to those reporting 30-day follow-up and to patients undergoing coronary artery bypass graft surgery with cardiopulmonary bypass.

**Conclusions:**

Preoperative intra-aortic balloon pump reduces perioperative and 30-day mortality in high-risk patients undergoing elective coronary artery bypass grafting.

**Electronic supplementary material:**

The online version of this article (doi:10.1186/s13054-014-0728-1) contains supplementary material, which is available to authorized users.

## Introduction

The intra-aortic balloon pump is used in different clinical conditions where myocardial function is diminished, even if its effect on outcome is debated; indeed, it does not improve 30-day and one-year survival in patients with acute coronary syndrome complicated by cardiogenic shock undergoing revascularization [1,2].

Its beneficial physiological effects are well recognized: the intra-aortic balloon pump acts by increasing diastolic blood pressure [3,4] and improving diastolic coronary perfusion. Furthermore, it increases cardiac output and stroke volume by reducing afterload. The ability to act on diastolic pressure has a great importance in clinical practice since the augmented diastolic pressure results in a redistribution of coronary blood flow toward ischemic areas of the myocardium [5,6].

The perioperative use of intra-aortic balloon pump in cardiac surgery is widespread and supported by a large amount of data [7,8]. Indeed, intra-aortic balloon pump use is among the few topics with a documented improvement in survival according to randomized evidence as summarized in a recent international consensus conference [9].

A recent randomized trial on intra-aortic balloon pump use in coronary artery bypass graft surgery was underpowered to confirm the beneficial effects of intra-aortic balloon pump on survival and concluded that in patients undergoing nonemergent coronary operations, with a stable hemodynamic profile and a left ventricular ejection fraction less than 35%, the preincision (after anesthesia induction and before skin incision) insertion of intra-aortic balloon pump did not result in a better outcome [10].

We therefore decided to perform an updated meta-analysis of randomized trials studying the effect of preoperative intra-aortic balloon pump use in coronary artery bypass graft surgery to address the effect of this therapy on survival.

## Materials and methods

We performed a systematic review and meta-analysis of randomized trials in accordance with the Preferred Reporting Items for Systematic reviews and Meta-Analyses (PRISMA) guidelines.

### Data sources and study selection

We searched for all randomized controlled trials, of any size or length of follow-up that reported the use of preoperative (before surgery) intra-aortic balloon pump in adult high-risk patients undergoing cardiac surgery. We defined as high risk the patients with at least one among: left ventricular ejection fraction <40%, left main coronary artery stenosis equal or more than 70%, reoperation, unstable angina despite medical treatment, recent acute myocardial infarction, or left ventricular hypertrophy in patients with coronary artery disease undergoing coronary artery bypass graft. Potentially eligible trials were identified by searching the Medline, Embase, Scopus, ISI Web of Knowledge and The Cochrane Library (updated 1 August 2014) using a combination of subject headings and text words to identify randomized controlled trials of preoperative intra-aortic balloon pump use. The full Medline search strategies aimed to include any randomized controlled trials ever performed on humans using preoperative intra-aortic balloon pump in any cardiac surgery is presented in Additional file 1. Searches were not restricted by language or publication status. We also examined the reference lists of eligible trials and reviews together with the abstracts of international congresses. We excluded duplicate publications, studies without mortality data, abstracts older than three years (those that did not reach the full peer review publication status) and those comparing different timing of intra-aortic balloon pump positioning (for example all patients were assigned to receive an intra-aortic balloon pump before or after surgery). Two authors independently screened the search output to identify records of potentially eligible trials, the full texts of which were retrieved and assessed for inclusion.

### Outcome data

The primary end point for the present meta-analysis was mortality at the longest follow-up as reported by authors (up to three months in these trials) and the secondary end point was 30-day mortality.

### Data extraction and risk of bias assessment

We extracted data on setting, outcome and length of follow-up. If a trial reported multiple comparisons, the comparators were either aggregated as a single group or excluded depending on the type of treatment and the specific analysis performed. We assessed the risk of bias associated with the method of sequence generation, allocation concealment, blinding, and the completeness of outcome data. We rated the risk of bias as being low, unclear, or high according to established criteria.

### Statistical analysis

To analyze the binary outcome we calculated the natural logarithms of risk ratios and its standard deviation. We pooled these using the inverse variance method and a fixed effect model [11]. To assess heterogeneity in results of individual studies, we used Cochran’s *Q* statistic and the I-square statistic (I-square >25% was used as a threshold indicating significant heterogeneity). Publication bias was assessed by visually inspecting funnel plots of the primary outcome, by analytical appraisal based on the Begg adjusted-rank correlation test and on Egger’s linear regression test (a two-sided *P* value of 0.10 or less was regarded as significant).

Sensitivity analyses were done to quantify the effect of intra-aortic balloon pump when restricted to trials with low risk of bias, to trials that report the 30-day mortality, and divided by on- and off-pump setting. We also investigated the influence of a single study on the overall risk estimate by sequentially removing a study to test the robustness of the main results.

Statistical significance was set at the two-tailed 0.05 level for hypothesis testing. Data analysis was performed using STATA 11.0 Software (StataCorp LP, College Station, TX, USA).

## Results

### Characteristics of the included individual studies

**Figure 1:**
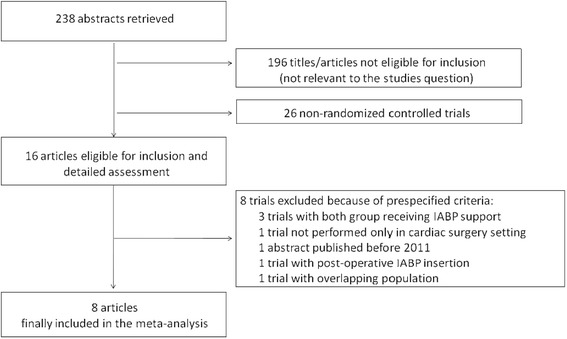
**Flow diagram for selection of articles.**

Out of 238 publications we selected eight randomized trials [10,12-18] meeting the inclusion/exclusion criteria (Figure 1) while another eight manuscripts were considered major exclusions [1,19-24] because all patients had received intra-aortic balloon pump support [19,20,24], the trial was not performed only in a cardiac surgery setting [1], the abstract was published before 2011 [23], the intra-aortic balloon pump was inserted postoperatively [22] and because of overlapping population [21,2].

The eight included trials randomized 625 patients (312 to the intra-aortic balloon pump group and 313 to the control group). Table 1 shows the clinical data on setting, type of comparator, outcome data and length of follow-up. Study quality appraisal indicated that the eight included studies were of variable quality (Table S1 in Additional file 1) and that only four of them had a low risk of bias.

**Table 1:** **Description of the eight randomized studies included in the meta-analysis**

**Author**	**Year**	**Journal**	**Setting**	**Time of insertion**	**Crossover**	**Complications**	**IABP patients**	**Control patients**	**Longest follow-up**
Ranucci *et al*. [8]	2013	*Crit Care Med*	CABG	After anesthesia induction and before skin incision	7/55	4 in IABP group	55	55	30 days
Lomivorotov *et al*. [15]	2012	*J Cardiothorac Vasc Anesth*	CABG	16 to 18 hours before surgery (in the ICU)	Not reported	Not reported	30	30	30 days
Shi *et al*. [16]	2011	*J Int Med Res*	OPCABG	1 hour before surgery	Not reported	Not reported	107	125	Hospital stay
Christenson *et al.* [14]	2003	*J Card Surg*	REDO-OPCABG	After anesthesia induction and before skin incision	7/15	0	15	15	2 months
Christenson *et al*. [13]	1999	*Ann Thorac Surg*	CABG	3 different groups:	23/30	2 in IABP group	30	30	Hospital stay
				−24 hours before surgery (in the ICU)		3 in crossovers			
				−12 hours before surgery (in the ICU)					
				−before anesthesia induction					
Christenson *et al*. [10]	1997	*Ann Thorac Surg*	REDO-CABG	Before anesthesia induction in theatre (4 patients had insertion in the ICU before transfer to the operating room)	9/24	1 in IABP group	24	24	3 months
						1 in crossover			
Christenson *et al*. [11]	1997	*Eur J Cardiothorac Surg*	CABG	2 different groups	11/20	0	32	20	Hospital stay
				−24 hours before surgery (in the ICU)					
				− preoperatively in the operating room, On average 1.5 hours before surgery					
Christenson *et al*. [12]	1997	*Thorac Cardiovasc Surg*	CABG	On average 2 h before surgery	Not reported	Not reported	19	14	3 months

Crossover: number of patients assigned to the no-IABP group who received postoperative IABP insertion because of low cardiac output syndrome. CABG: coronary artery bypass graft surgery; ICU: intensive care unit; OPCABG: off-pump coronary artery bypass graft surgery; IABP: intra-aortic balloon pump; REDO: a patient who had already undergone sternotomy for cardiac surgery once in the past.

### Quantitative data synthesis

**Figure 2:**
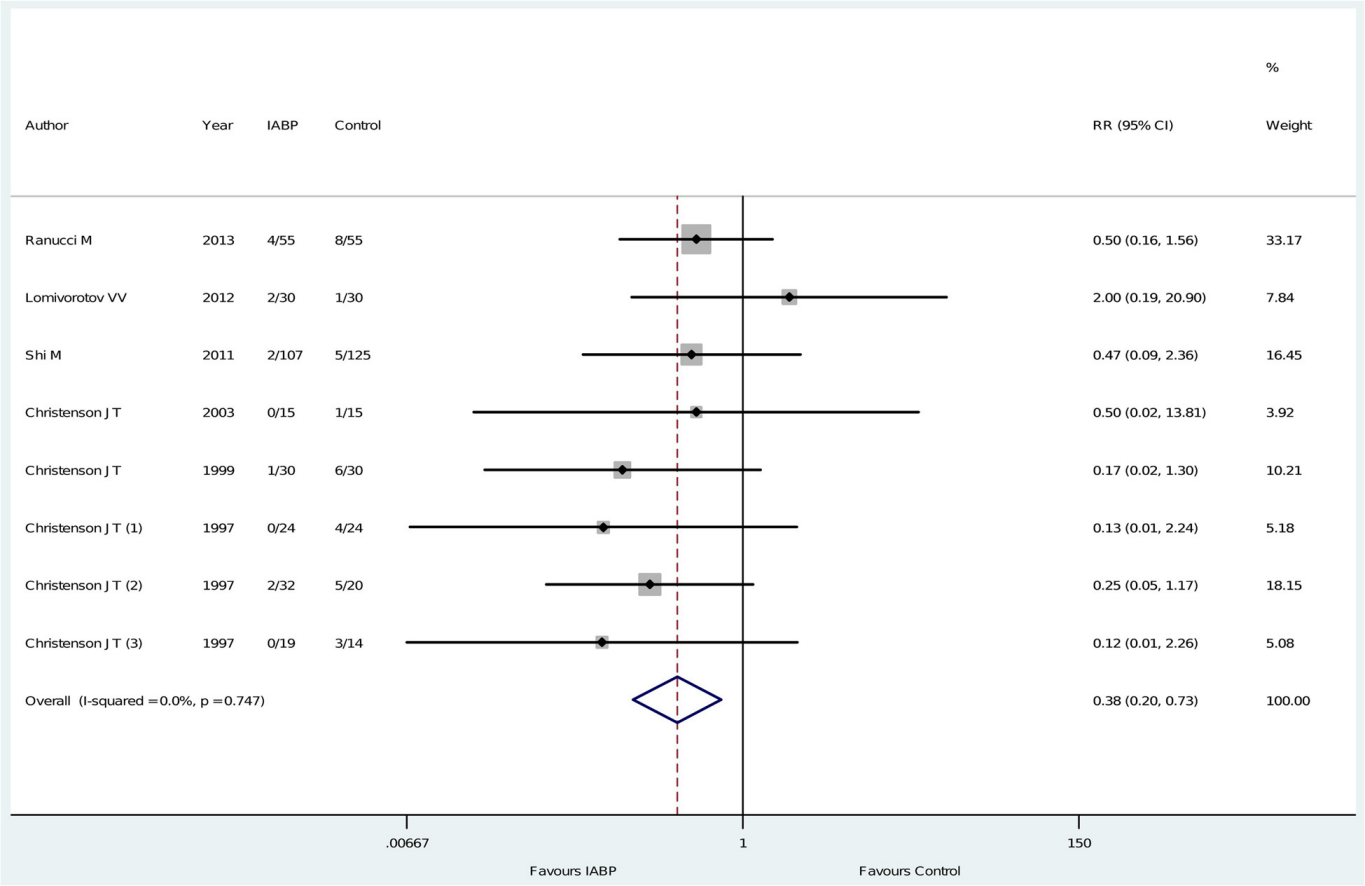
**Forest plot for the risk of overall mortality.** RR: relative risk; CI: confidence interval.

**Figure 3:**
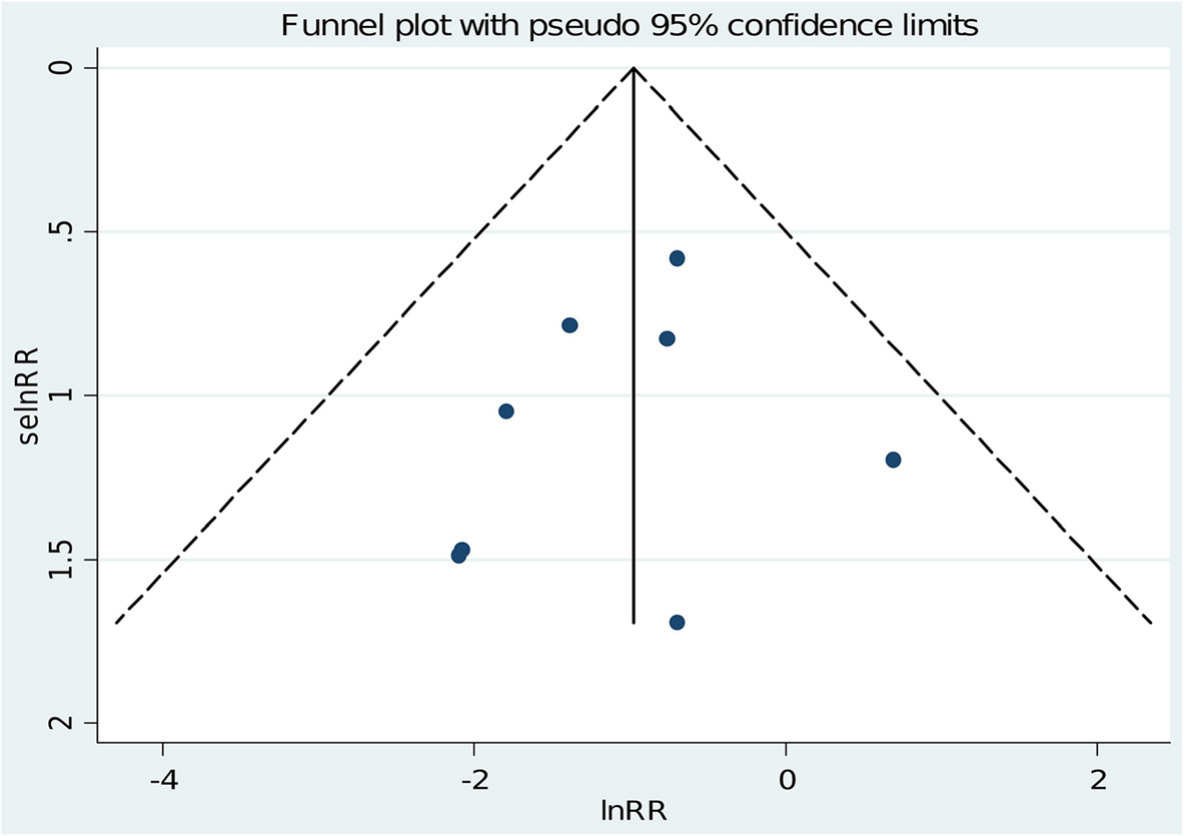
**Funnel plot for the risk of mortality.** LnRR: natural logarithm of the risk ratio; selnRR: standard error of the natural logarithm of the risk ratio.

**Figure 4:**
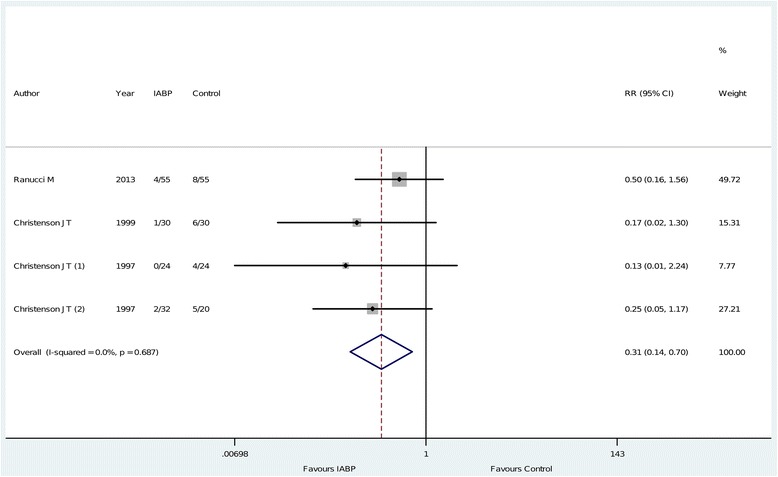
**Forest plot for the risk of mortality in low risk of bias trials.** IABP: intra-aortic balloon pump; RR: risk ratio; CI: confidence interval.

**Figure 5:**
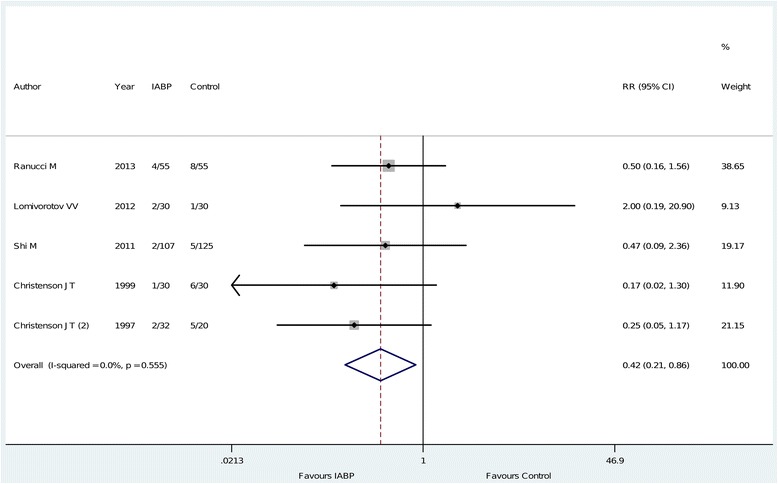
**Forest plot for the risk of 30-day mortality.** IABP: intra-aortic balloon pump; RR: risk ratio; CI: confidence interval.

The principal analysis (Figure 2) showed that the use of intra-aortic balloon pump was associated with a significant reduction in the risk of overall mortality (11 of 312 (3.5%) in the intra-aortic balloon pump group versus 33 of 313 (11%) in the control group, risk ratio = 0.38 (0.20 to 0.73), *P* for effect = 0.004, *P* for heterogeneity = 0.7, I-square = 0%, with eight studies included). Visual inspection of funnel plots did not identify a skewed or asymmetrical shape (Figure 3) and quantitative evaluation did not suggest a presence of publication bias, as measured by Egger test (*P* = 0.6) and Begg test (*P* = 0.5). Sensitivity analyses considering only data from the four studies with low risk of bias (Figure 4) confirmed the reduction in the risk of mortality (7 of 141 (5.0%) in the intra-aortic balloon pump group versus 23 of 129 (18%) in the control group, risk ratio = 0.31, (0.14 to 0.70), *P* for effect = 0.005, *P* for heterogeneity = 0.7, I-square = 0%, with four studies included). The overall result was also confirmed restricting the analysis to trials that reported the 30-day mortality (Figure 5) (11 of 254 (4.3%) in the intra-aortic balloon pump group versus 25 of 260 (9.6%) in the control group, risk ratio = 0.42 (0.21 to 0.86), *P* for effect = 0.02, *P* for heterogeneity = 0.6, I-square = 0%, with six studies included) and in surgeries performed with cardiopulmonary bypass (9 of 190 (4.7%) in the intra-aortic balloon pump group versus 27 of 173 (16%) in the control group, risk ratio = 0.36 (0.17 to 0.74), *P* for effect = 0.01, *P* for heterogeneity = 0.5, I-square = 0%, with six studies included) (Additional file 2: Figure S1).

Moreover, sensitivity analyses performed by removing each single study from the meta-analysis to determine the influence of individual data sets to the pooled risk ratios (Additional file 3: Figure S2), did not determine major changes in direction or magnitude of statistical findings, confirming for each analysis the pooled effect (risk ratio <1) and the statistical significance (*P* <0.05).

## Discussion

The most important finding of this meta-analysis is that preoperative insertion of intra-aortic balloon pump reduces mortality in elective high-risk coronary artery bypass graft patients. This finding was already suggested by previous meta-analyses [7,8,25] but they were challenged by a recent large randomized controlled trial that concluded that the intra-aortic balloon pump was not useful in this context even if it showed a trend toward improved clinically relevant outcomes (major morbidity: 22 (40%) in the intra-aortic balloon pump group versus 17 (31%) in the control group, odds ratio =1.49 (0.68 to 3.33), *P* = 0.3, operative mortality: 4 (7.3%) in the intra-aortic balloon pump group versus 8 (14%) in the control group, odds ratio = 0.46 (0.13 to 1.43), *P* = 0.2).

When compared with other meta-analyses our work includes three recent articles for a total of 402 new patients. We also included sensitivity analyses, almost double the number of patients with respect to previous meta-analyses and, most importantly, our meta-analysis includes articles from four different research groups from four different countries.

The role of the intra-aortic balloon pump has been recently challenged by Thiele’s study [1] that showed no difference in short- and long-term survival in patients randomized to intra-aortic balloon pump versus standard treatment (risk ratio = 2.60 (0.95 to 7.10), *P* = 0.05), recurrent revascularization (risk ratio = 0.91 (0.58 to 1.41), *P* = 0.77), or stroke (risk ratio = 1.50 (0.25 to 8.84), *P* = 1.00) in acute coronary syndrome complicated by cardiogenic shock and planned for revascularization. It should be noted that that study is not included in this meta-analysis because only six patients underwent surgical revascularization.

The intra-aortic balloon pump acts by increasing diastolic blood pressure [3], directly improving diastolic coronary perfusion and increasing cardiac output and stroke volume by reducing afterload. The ability to act on diastolic pressure has a great importance in clinical practice because previous studies have demonstrated that the augmented diastolic pressure results in a redistribution of coronary blood flow toward ischemic areas of the myocardium [5,6].

The value of some trials is to be questioned in the light of the unacceptably high rate of intra-aortic balloon pump-related complications [26,27]. The most recent trial [10] reported two patients in the intra-aortic balloon pump study group who did not receive the balloon due to technical difficulties and four patients suffering from vascular complications (12%). Strikingly, both in the surgical [10] and nonsurgical [2] population of previously published studies there was a systematic need for crossover from the control group to the intra-aortic balloon pump group (approximately 13 to 17%), which assumes a reproducible methodological bias.

### Limitations

The main limitation of the present meta-analysis is that four out of the eight included randomized controlled trials were of suboptimal quality (but the results were confirmed when analyzing only the trials with low risk of bias). Furthermore, traditional limitations of meta-analyses due to variations in the treatment regimens, in populations or major subgroups within trials, and in the conduct of the trials apply to this study [28,29]. The possibility of the beneficial effect of intra-aortic balloon pump in non-isolated coronary operations is still to be investigated since worldwide there is an increased percentage of associated procedures and some trials [10] do not specify this surgical data.

## Conclusions

Prophylactic intra-aortic balloon pump use reduces perioperative mortality in high-risk patients undergoing cardiac surgery according to the most updated evidence-based medicine.

## Key messages

- Intra-aortic balloon pump is used in different clinical conditions where myocardial function is diminished.
- Intra-aortic balloon pump acts by increasing diastolic blood pressure with consequent redistribution of coronary blood flow toward ischemic areas of the myocardium.
- Intra-aortic balloon pump increases cardiac output and stroke volume by reducing afterload.
- Recent evidence challenges the use of intra-aortic balloon pump after myocardial infarction.
- Preoperative insertion of intra-aortic balloon pump reduces mortality in elective high-risk coronary artery bypass graft patients.

## Additional files

Additional file 1:
**Medline search strategy and methodological quality summary.**
Additional file 2: Figure S1. Forest plot for the risk of overall mortality in on-pump and off-pump setting. Additional file 3: Figure S2. Sensitivity analyses showing the risk ratio estimate and 95% confidence interval omitting one study at a time.
