# Supplementary material for: Preoperative intra-aortic balloon pump to reduce mortality in coronary artery bypass graft: a meta-analysis of randomized controlled trials
Source: Crit Care. 2015 Jan 14;19(1):10. doi: 10.1186/s13054-014-0728-1 (PMC4316767; doi:10.1186/s13054-014-0728-1)
Supplement: Additional file 1: — Medline search strategy and methodological quality summary. [file 13054_2014_728_MOESM1_ESM.docx]

**SUPPLEMENTARY MATERIAL**

**APPENDIX 1: MEDLINE SEARCH STRATEGIE.**

((IABP[ti] OR "Intra-Aortic Balloon Pumping"[ti] OR "Intra-Aortic Balloon Pump"[ti]) AND (randomized controlled trial[pt] OR controlled clinical trial[pt] OR randomized controlled trials[mh] OR random allocation[mh] OR double-blind method[mh] OR single-blind method[mh] OR clinical trial[pt] OR clinical trials[mh] OR (clinical trial[tw] OR ((singl*[tw] OR doubl*[tw] OR trebl*[tw] OR tripl*[tw]) AND (mask*[tw] OR blind[tw])) OR (latin square[tw]) OR placebos[mh] OR placebo*[tw] OR random*[tw] OR research design[mh:noexp] OR follow-up studies[mh] OR prospective studies[mh] OR cross-over studies[mh] OR control*[tw] OR prospectiv*[tw] OR volunteer*[tw])) NOT (animal[mh] NOT human[mh]))

**Supplemental Table 1:** Methodological quality summary: Review authors’ judgments about each methodological quality item for each included study.

| **TRIAL** | **ENTRY** | **JUDGEMENT** | **DESCRIPTION** |
| --- | --- | --- | --- |
| Ranucci M 2013 | Adequate sequence generation? | YES | Electronically generated. |
|  | Allocation concealment? | YES | Sealed numbered envelops. |
|  | Blinding of participants and personnel? | NO | The presence of IABP precludes blinding. |
|  | Blinding of outcome assessment? | UNCLEAR | The manuscript doesn't report information about the bliding of outcome assessment. |
|  | Complete outcome data addressed? | YES | The analysis was performed on the Intention To Treat population. |
|  | Free of selective reporting? | YES | All outcomes reported. |
|  | Free of other bias? | NO | Quote: “This study is limited to patients undergoing coronary operations with or without additional procedures (valve surgery, left ventricle reshaping) under stable hemodynamic conditions, having a poor LVEF as the inclusion criteria. We cannot therefore exclude that other kind of patient selection (additional risks like redo operation, left main stem occlusion, unstable angina) may result in a different patient selection, leading to different results. These additional risk factors are represented in our patient population, but subgroup analyses are not possible due to the relatively limited patient population.”, “ The authors also observed that each of the RCTs included a limited amount of patients and was funded in part by an industrial partner who might benefit from positive results.” |
|  | OVERALL RISK OF BIAS | LOW |  |
| Lomivorotov VV 2012 | Adequate sequence generation? | YES | Method of randomisation not stated. |
|  | Allocation concealment? | YES | Quote: "Randomization was performed using random words (IABP, IABP + Levo, or Levo) inserted into envelopes. Sealed envelopes were opened by the cardiologist one day before surgery" |
|  | Blinding of participants and personnel? | NO | The presence of IABP precludes blinding. |
|  | Blinding of outcome assessment? | UNCLEAR | The manuscript doesn't report information about the bliding of outcome assessment. |
|  | Complete outcome data addressed? | UNCLEAR | There aren't sufficient information to permit the judgement. |
|  | Free of selective reporting? | UNCLEAR | There aren't sufficient information to permit the judgement. |
|  | Free of other bias? | NO | This study has included a highly select group of patients and the distribution of women and men is not homogenous in the groups. |
|  | OVERALL RISK OF BIAS | MODERATE |  |
| Shi M 2011 | Adequate sequence generation? | YES | Patients were randomly divided into two groups according to a computer generated randomization schedule. |
|  | Allocation concealment? | UNCLEAR | There aren't sufficient information to permit the judgement. |
|  | Blinding of participants and personnel? | NO | The presence of IABP precludes blinding. |
|  | Blinding of outcome assessment? | UNCLEAR | The manuscript doesn't report information about the bliding of outcome assessment. |
|  | Complete outcome data addressed? | UNCLEAR | There aren't sufficient information to permit the judgement. |
|  | Free of selective reporting? | UNCLEAR | There aren't sufficient information to permit the judgement. |
|  | Free of other bias? | NO | This study has included a highly select group of patients who have been randomised within the study but no blinding exists. |
|  | OVERALL RISK OF BIAS | MODERATE |  |
| Christenson JT (5) 2003 | Adequate sequence generation? | UNCLEAR | Randomisation process not described. |
|  | Allocation concealment? | UNCLEAR | Concealment method not stated |
|  | Blinding of participants and personnel? | NO | The presence of IABP precludes blinding. |
|  | Blinding of outcome assessment? | UNCLEAR | The manuscript doesn't report information about the bliding of outcome assessment. |
|  | Complete outcome data addressed? | YES | No incomplete data. |
|  | Free of selective reporting? | YES | All outcome data reported. |
|  | Free of other bias? | NO | This study has included a highly select group of patients (High-Risk OPCAB Surgery) |
|  | OVERALL RISK OF BIAS | MODERATE |  |
| Christenson JT (4) 1999 | Adequate sequence generation? | YES | Quote: "On admission to hospital the patients were randomly assigned to groups by lottery principle drawing pre-prepared sealed envelopes containing the group assignment" |
|  | Allocation concealment? | YES | Sealed envelopes. |
|  | Blinding of participants and personnel? | NO | The presence of IABP precludes blinding. |
|  | Blinding of outcome assessment? | UNCLEAR | The manuscript doesn't report information about the bliding of outcome assessment. |
|  | Complete outcome data addressed? | YES | No incomplete data. |
|  | Free of selective reporting? | YES | All outcomes reported. |
|  | Free of other bias? | NO | This study has included a highly select group of patients (High-Risk coronary patients). |
|  | OVERALL RISK OF BIAS | LOW |  |
| Christenson JT (1) 1997 | Adequate sequence generation? | YES | Quote: "On admission to the hospital, the patients were randomised by lottery principle, drawing pre-prepared sealed envelopes containing the group assignment" |
|  | Allocation concealment? | YES | Sealed envelopes. |
|  | Blinding of participants and personnel? | NO | The presence of IABP precludes blinding. |
|  | Blinding of outcome assessment? | UNCLEAR | The manuscript doesn't report information about the bliding of outcome assessment. |
|  | Complete outcome data addressed? | YES | No missing outcome data. |
|  | Free of selective reporting? | YES | All outcomes reported. |
|  | Free of other bias? | NO | This study has included a highly select group of patients (Redo CABG patients). |
|  | OVERALL RISK OF BIAS | LOW |  |
| Christenson JT (2) 1997 | Adequate sequence generation? | YES | Quote: "The patients were randomised into either of three groups by lottery" |
|  | Allocation concealment? | YES | Sealed envelopes. |
|  | Blinding of participants and personnel? | NO | The presence of IABP precludes blinding. |
|  | Blinding of outcome assessment? | UNCLEAR | The manuscript doesn't report information about the bliding of outcome assessment. |
|  | Complete outcome data addressed? | YES | No incomplete data. |
|  | Free of selective reporting? | YES | All outcomes reported. |
|  | Free of other bias? | NO | This study has included a highly select group of patients (high risk coronary patients). |
|  | OVERALL RISK OF BIAS | LOW |  |
| Christenson JT (3) 1997 | Adequate sequence generation? | UNCLEAR | Randomisation process not described. |
|  | Allocation concealment? | UNCLEAR | Concealment method not described. |
|  | Blinding of participants and personnel? | NO | The presence of IABP precludes blinding. |
|  | Blinding of outcome assessment? | UNCLEAR | The manuscript doesn't report information about the bliding of outcome assessment. |
|  | Complete outcome data addressed? | YES | No missing outcome data. |
|  | Free of selective reporting? | YES | All outcomes reported. |
|  | Free of other bias? | NO | This study has included a highly select group of patients (patients with coronary artery disease, poor left ventricular function (LVEF < 40%, hypertensive LV Hypertrophy ). |
|  | OVERALL RISK OF BIAS | MODERATE |  |
