# Supplementary figures and images for: Preoperative intra-aortic balloon pump to reduce mortality in coronary artery bypass graft: a meta-analysis of randomized controlled trials
Source: Crit Care. 2015 Jan 14;19(1):10. doi: 10.1186/s13054-014-0728-1 (PMC4316767; doi:10.1186/s13054-014-0728-1)

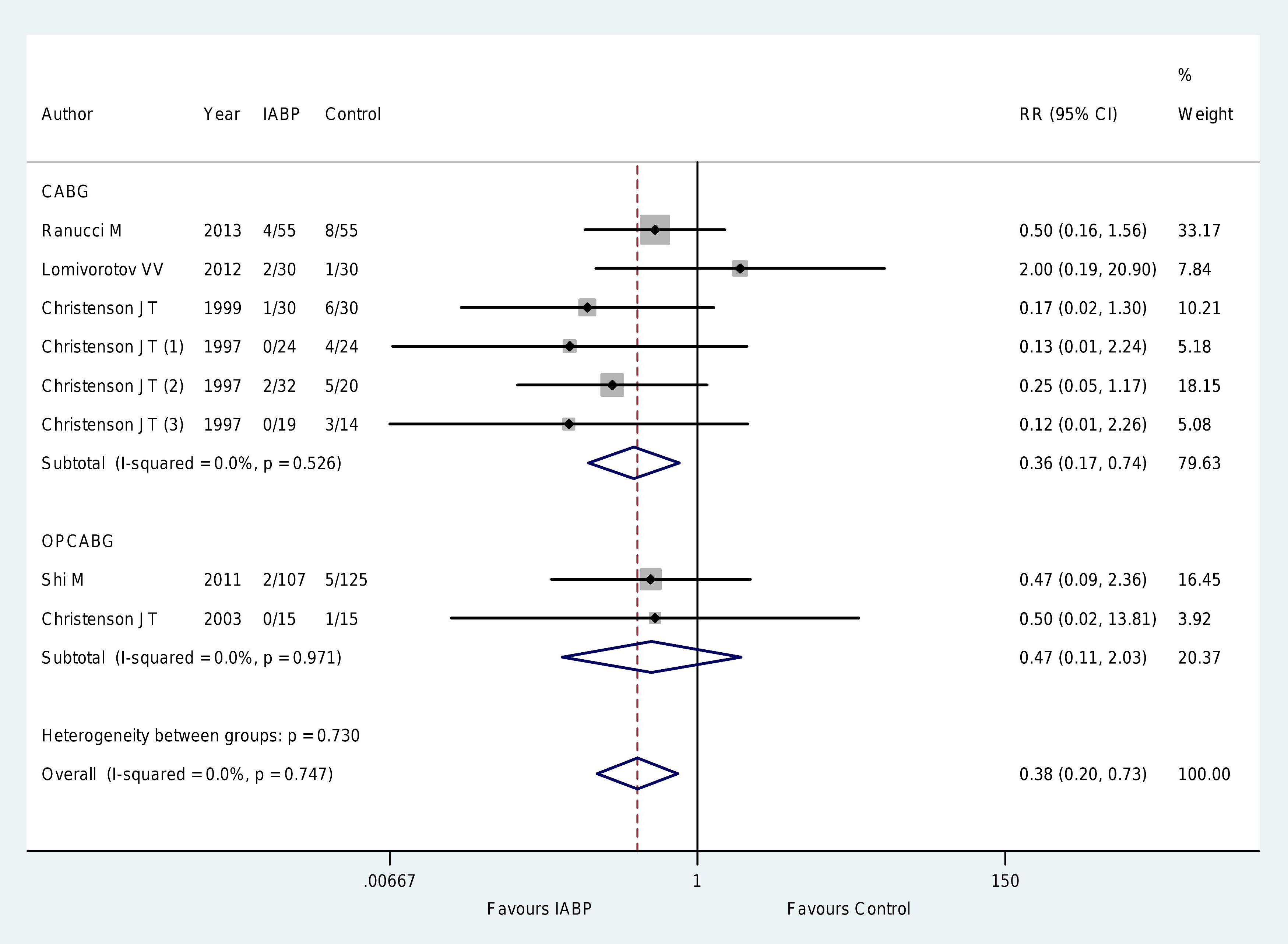

Supplement: Additional file 2: Figure S1. — Forest plot for the risk of overall mortality in on-pump and off-pump setting. [file 13054_2014_728_MOESM2_ESM.jpeg]

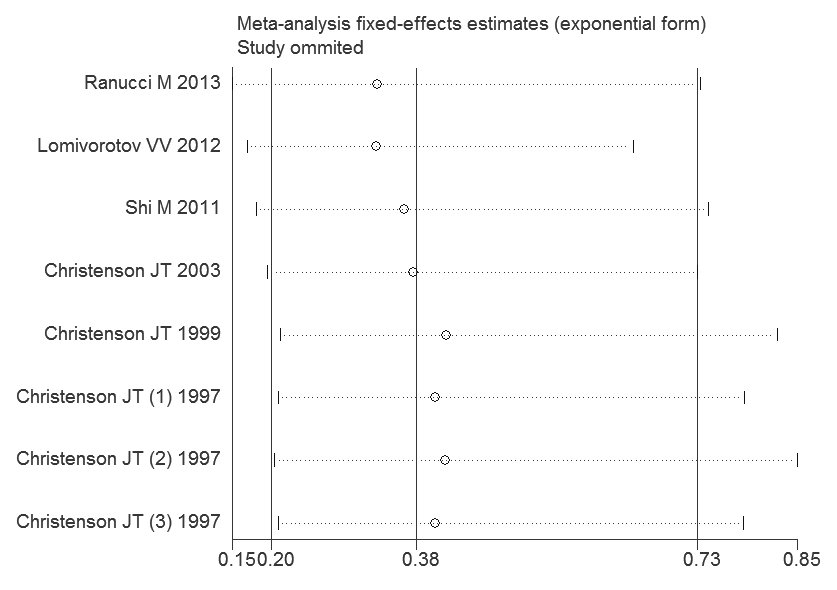

Supplement: Additional file 3: Figure S2. — Sensitivity analyses showing the risk ratio estimate and 95% confidence interval omitting one study at a time. [file 13054_2014_728_MOESM3_ESM.jpeg]
